# Sit to Talk: Relation between Motor Skills and Language Development in Infancy

**DOI:** 10.3389/fpsyg.2016.00475

**Published:** 2016-03-31

**Authors:** Klaus Libertus, Dominic A. Violi

**Affiliations:** Department of Psychology, Learning Research and Development Center, University of PittsburghPittsburgh, PA, USA

**Keywords:** motor development, language development, developmental cascades, infancy, videoconference

## Abstract

Relations between walking skills and language development have been reported in 10- to 14-month-old infants. However, whether earlier emerging motor milestones also affect language skills remains unknown. The current research fills this gap by examining the relation between reaching and sitting skills and later language development, respectively. Reaching and sitting were assessed eight times, starting when infants (*N* = 29) were around 3 months of age. All assessments were completed and recorded remotely via videoconference using Skype or FaceTime. Subsequently, infants’ language and motor skills were assessed via parent questionnaires (Communicative Development Inventories and Early Motor Questionnaire) at 10 and 14 months of age. Results revealed a significant correlation between the emergence of sitting skills and receptive vocabulary size at 10 and 14 months of age. Regression analyses further confirmed this pattern and revealed that the emergence of sitting is a significant predictor of subsequent language development above and beyond influences of concurrent motor skills. These findings suggest that the onset of independent sitting may initiate a developmental cascade that results in increased language learning opportunities. Further, this study also demonstrates how infants’ early motor skills can be assessed remotely using videoconference.

## Introduction

Motor skills are at the core of infants’ (and adults’) everyday actions and interactions and consequently affect subsequent perceptual, cognitive, and social development (Gibson, 1988; Bushnell and Boudreau, 1993). Piaget (1952) suggested a relation between motor and cognitive development and noted that infants’ own actions and resulting sensorimotor experiences are critical for their learning about the environment and the objects within it. Since Piaget’s original observations, several studies have reported evidence for relations between motor skills and development in seemingly unrelated domains – such as object perception, face processing, and language skills. For example, object exploration skills have been found to facilitate object segregation abilities in 3-month-old infants (Needham, 2000). At the same age, early experiences of successful reaching have been found to encourage infants’ attention to faces over objects (Libertus and Needham, 2011). Similarly, the onset of independent sitting around 5–7 months has been associated with improved 3-D object completion (Soska et al., 2010) and a temporary disruption in infant’s holistic face processing skills (Cashon et al., 2013). Finally, two studies have reported associations between the onset of independent walking and language development in 10- to 14-month-old infants, with walking infants showing larger vocabularies than crawling infants (Walle and Campos, 2014; He et al., 2015). Together, these findings demonstrate that the acquisition of a new motor skill (e.g., reaching, sitting, or walking) has consequences for infants’ concurrent abilities in the perceptual, cognitive, or social domains. The current study aims to longitudinally examine the *predictive* relations between motor skills emerging in early infancy and later language development.

To date, a number of studies have examined associations between motor and communicative development in children with developmental disorders such as autism spectrum disorder (ASD). Children with ASD often show impaired language abilities, but earlier emerging motor difficulties have also been documented (Teitelbaum et al., 1998; Provost et al., 2007; Lloyd et al., 2013). For example, infants at high familial risk for ASD (HR infants, who have an older sibling with ASD diagnosis) show reduced fine motor and grasping skills at age 6 months (Libertus et al., 2014) and delayed development of posture skills (i.e., sitting and standing) between six to 14 months (Nickel et al., 2013). Consequently, motor delays during the first 2 years of life have been hypothesized to affect subsequent social development and may contribute to language delays in children with ASD (Bhat et al., 2011). Empirical evidence supports this hypothesis with findings suggesting that fine motor skills between 12- to 18 months and at 24 months predict expressive language skills at 36 months of age in HR infants (LeBarton and Iverson, 2013).

Early motor delays have also been noted in other developmental disorders such as Specific Language Impairment (Hill, 2001; for review see Leonard and Hill, 2014). Consequently, it is possible that early motor development predicts subsequent language development in typically developing children as well. However, only few studies have examined motor and language associations longitudinally in typically developing infants. For example, parent report on the onset of independent sitting and walking was found to predict infants’ productive vocabulary between 16 to 28 months (Oudgenoeg-Paz et al., 2012). Further, a large-scale cohort study of 62,944 children found that motor skills at 18 months were predictive of *subsequent* language skills at 36 months of age (Wang et al., 2014). These findings demonstrate that the mastery of certain motor milestones seem to be related to subsequent language development. However, before the mastery of a new skill comes a period of trial and error. Whether infants’ transition through this *unstable acquisition period* is related to later language learning is unknown. The current study fills this gap in the literature by longitudinally following infants during their first attempts with two foundational motor skills: grasping objects and sitting independently.

Grasping and sitting skills emerge very early in life and enable infants to actively interact with the physical and social world (Libertus, 2010). Independent sitting facilitates visual and manual exploration of the environment by changing the child’s point-of-view and freeing their hands for manual exploration (Rochat and Goubet, 1995; Harbourne et al., 2013). Successful grasping enables the infant to obtain and explore objects, resulting in new opportunities to learn about object features and functions (Lederman and Klatzky, 2009). Both skills are likely to have lasting impacts on children’s subsequent development. For example, providing 3-month-old infants with scaffolded reaching experiences using Velcro mittens and toys facilitates immediate grasping skills (Needham et al., 2002; Libertus and Needham, 2010; Libertus and Landa, 2014) as well as object exploration and attention-focusing skills 1 year after the original training sessions (Libertus et al., 2015). Further, more active exploration at 5 months of age has been related to higher intellectual functioning at 4 and 10 years of age (Bornstein et al., 2013). One likely explanation for these findings is that new motor skills change how infants interact with objects, how they interact with people, and potentially also how people respond to them. Such changes have been observed with regard to locomotion, where crawling infants elicit different verbal responses from their parents than walking infants (Karasik et al., 2014). Consequently, it is likely that the acquisition of sitting and grasping also results in new learning opportunities, which may in turn facilitate the development in other domains such as language learning.

The current study examines the emergence of sitting and reaching skills between three to 5 months of age and their relation to subsequent language development at 10 and 14 months of age in typically developing infants. Previous studies have reported associations between motor skills emerging during the 2nd year and language at 3 years of age (LeBarton and Iverson, 2013; Wang et al., 2014). Based on these findings, we hypothesize to find a similar relation between infants’ transition into reaching and sitting and their subsequent language skills at 10 and possibly at 14 months of age. In addition, concurrent motor skills have been found to predict language skills in 10- to 14-month-olds (Walle and Campos, 2014; He et al., 2015). Therefore, we will also examine the role of concurrent motor skills at 10 and 14 months on the associations between early motor and later language development. Finally, the current study is the first to remotely record high-density behavioral data on infants’ early motor development using videoconferencing. The potential of this new method for future applications will be examined and discussed. While the relation between sitting and grasping development is also of great theoretical interest, this topic will not be covered here and instead will be discussed in a separate report.

## Materials and Methods

### Participants

Participants were 29 full-term infants (*M* = 39.79 weeks gestation, *SD* = 1.18) who were recruited between three to 4 months of age using social media posts. Families were located in eight different U.S. states (including Washington D.C.), were highly educated, and completed assessments remotely via videoconferencing and online questionnaires. All families completed a follow-up assessment when their child was 10 months of age, and 24 families (83%) completed a follow-up assessment when their child turned 14 months of age. Two additional families were recruited into the study but dropped out after two assessments or failed to complete the follow-up questionnaires. See **Table 1** for details about the final sample. Parents of all participants provided both recorded verbal and online consent. Procedures followed ethical guidelines and were approved by the Institutional Review Board at the University of Pittsburgh.

**Table 1:** Participant characteristics.

N	Age at study onset	Age at 10 month follow-up	Age at 14 month follow-up^∗^	#F	Weight at birth (Grams)	Location (US state)	Parent education	Race
29	3.55 (0.27)	10.07 (0.26)	14.15 (0.47)	14	3419.54 (440.98)	2 CA; 3 DC; 1 MA; 4 MD; 1 MS; 3 NY; 13 PA; 1 TX	8.03 (1.55)	1 A, 25 C, 3 M

Counts are provided for the total number of participants (N), the number of female participants (#F), the state of residence, and racial composition of the sample. All other values are group averages with standard deviations in parentheses. Ages are reported in months. Parents’ education level was assessed on a scale from 0 (no High School degree) to 5 (Doctorate degree) for each parent and summed (maximum 10). Race abbreviations: C, Caucasian, A, Asian, M, more than one race. *only 24 families completed the 14-month follow-up.

### Procedure

Starting around 3 months of age, all infants completed eight weekly sitting and grasping assessments (described below). All assessments took place in the child’s own home and were recorded via video chat using the family’s own camera-equipped computer, phone, or tablet (18 families used Microsoft Skype, 11 families used Apple FaceTime). The live video feed from the participants was captured and stored at a resolution of 1280 × 720 pixel using a video capture device (Elgato Game Capture HD). Before study onset, the experimenter demonstrated all procedures via videoconference using a life-sized baby doll. This step was included to show parents the optimal positioning of their video camera during the sessions. To minimize distraction during the videoconference sessions, parents were also instructed to maximize their application window while the camera of the experimenter was covered with black tape – resulting in a mostly black screen in front of the infant. Each video-chat session lasted about 5–8 min and the eight assessments were approximately 1 week apart for all families (*M* = 6.93 days, *SD* = 0.18).

At around 10 and 14 months of age, infants’ parents received an email inviting them to complete two follow-up online questionnaires about their child’s motor and communicative development (see below). An online survey system (Qualtrics) was used to collect questionnaire responses.

### Measures

#### Sitting Task

**FIGURE 1:**
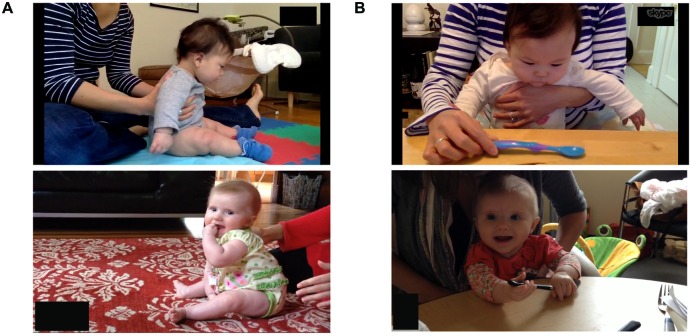
**Examples of the sitting task (A) and the grasping task (B)**. Parental consent to use the photos of the participants has been obtained.

Infants’ ability to sit independently was measured during a 1-min observation where the child was placed into a self-sitting posture on the floor. Parents were instructed to sit behind the child and to provide initial postural support before removing the support and allowing the child to attempt independent sitting. If the child lost balance, parents caught the child before falling and placed the child back into the starting position for another attempt. During this assessment, the camera was placed to the side to provide a profile view of the infant (see **Figure 1A**). Trained observers coded all videos frame-by-frame using spine and arm positions to categorize infants’ posture as either “sitting” (spine maintained at above 45° angle to floor with or without arm support) or “not sitting” (spine below 45° angle to floor, lying on tummy, or supported by parent). For analyses, the total duration spent in the “sitting” posture was calculated as a proportion of the total trial duration. Each infant completed the sitting task eight times, resulting in a total of 232 sitting task videos that were coded. Inter-rater reliability was assessed on 141 (61%) randomly selected videos and was good (Intraclass Correlation Coefficient = 0.86).

#### Grasping Task

Infants’ ability to grasp an object was measured during a 1-min observation where the child was seated on a parent’s lap at a table. Parents were instructed to place a spoon on the table in front of the child, tap, and lift the spoon briefly, and verbally encourage reaching for the spoon throughout the trial. During this assessment, the camera was placed across from the child to provide a frontal view of the infant’s face and arms (see **Figure 1B**). Trained observers coded all videos frame-by-frame, recording the action of each hand separately. Grasping was defined as any manual contact with the spoon that resulted in a partial or complete lifting of the spoon. This definition of grasping focuses on action *consequences* (i.e., spoon off the table) rather than means and is appropriate for young infants (Libertus and Needham, 2014). For analyses, the total grasping duration was calculated as a proportion of the total trial duration. Each infant completed the grasping task eight times, resulting in a total of 232 grasping task videos that were coded. Inter-rater reliability was assessed on 86 (37%) randomly selected videos and was excellent (Intraclass Correlation Coefficient = 0.96).

#### Early Motor Questionnaire (EMQ)

The Early Motor Questionnaire (EMQ) is a parent-report measure of motor skill development in children between 2 to 24 months of age. Parents answer simple questions about their child’s motor abilities in everyday contexts, including 49 gross motor skills, 48 fine motor skills, and 31 perception-action skills (total 128 questions). Parents rate each motor skill on a 5-point scale, ranging from -2 (sure child does NOT show skill) to +2 (sure child shows skill). Raw scores are calculated by summing all responses and were used for analysis in the current study (ranging from -256 to +256). The EMQ shows good validity in comparison to standardized, experimenter-administered motor assessments (for a copy of the EMQ see Supplemental Materials; for details on EMQ construction and validity see Libertus and Landa, 2013).

#### Communicative Development Inventories Words and Gestures (CDI)

The Communicative Development Inventories (CDI) is a parent-report questionnaire assessing children’s receptive and expressive vocabulary and is appropriate for children between 8 and 16 months of age (Fenson et al., 2006). It includes a 396-item vocabulary checklist plus a list of 31 familiar words and phrases the child may understand. Due to the young age of our participants, only receptive vocabulary raw scores were analyzed in the current study. To further increase variability in the receptive language scores, the vocabulary checklist was combined with the familiar words and phrases sections – resulting in a total possible score of 427.

### Analyses

Graphical and statistical examination of CDI raw scores at 10 months showed significant deviations from normality (*p* < 0.001, Shapiro–Wilk). Consequently, CDI scores were log-transformed prior to analysis. Graphical and statistical examination showed that the transformed CDI scores were normally distributed (*p*s > 0.29, Shapiro–Wilk). No other violations of normality were observed (*p*s > 0.15). Further, all variables were examined for extreme observations (defined as values of 3 + SDs above or below the group mean) but no outlying values were observed.

Development of grasping and sitting between 3–5 months of age and potential influences of Gender were first examined using repeated measures Multivariate Analysis of Variance (MANOVA) with Visit (8) as within-subjects factor and Gender (2) as between-subjects factor. Rate of growth over the eight assessments was then calculated for both grasping and sitting. The resulting grasping and sitting slopes quantify rate of skill acquisition rather than a static end state or skill onset point. Grasping and sitting slopes were used in correlation analyses to examine their relation with receptive language skills (CDI) at both 10 and 14 months of age. Significant correlations between early motor and later language scores were then followed up by regression analyses to examine predictive relations between early motor and later language skills above and beyond influences of concurrent motor scores obtained on the EMQ. Robust regression (Rousseeuw et al., 2015) was used for these follow-up analyses. Preliminary and correlation analyses were conducted using SPSS while regression analyses were conducted using R.

## Results

### Preliminary Analyses

A Visit (8) by Gender (2) MANOVA revealed a significant effect of Visit, *F*(14,15) = 17.02, *p* < 0.001, = 0.941, but no effect of Gender and no interaction (*p*s > 0.275). Separate follow-up ANOVAs (Greenhouse–Geisser corrected) for grasping and sitting revealed that both grasping, *F*(5.15, 196) = 19.09, *p* < 0.001, $\eta_{p}^{2}$ = 0.405, and sitting, *F*(4.60, 196) = 12.74, *p* < 0.001, $\eta_{p}^{2}$ = 0.313, durations increased significantly across the eight assessments. Within-subject contrasts in these models revealed significant *linear* developmental trends across visits for both grasping, *F*(1,28) = 111.57, *p* < 0.001, $\eta_{p}^{2}$ = 0.799, and sitting skills over time, *F*(1,28) = 54.14, *p* < 0.001, $\eta_{p}^{2}$ = 0.659. No higher order trends were significant (*p*s > 0.19). Consequently, linear slopes were calculated by taking grasping and sitting durations over the child’s chronological age at each of the eight assessments. This approach results in one grasping and one sitting slope capturing the individual rate of change for these two skills over the eight study visits for each participant. Comparisons between grasping (*M* = 1.19, *SD* = 0.60) and sitting slopes (*M* = 0.58, *SD* = 0.42) reveal overall faster increases in grasping than in sitting durations between 3–5 months of age, *t*(28) = 4.35, *p* < 0.001, 95% *CI* (0.32, 0.90). Relations between grasping and sitting development will be the focus of a future report and are not discussed in detail here.

### Correlation Analyses

The relation between growth in grasping and sitting skills from three to 5 months of age (i.e., slopes) and language and motor skills at 10 and 14 months of age was examined using Pearson correlation. Significant positive correlations were observed between sitting slopes and receptive language scores at both 10 months (*r*_29_ = 0.40, *p* = 0.029) and 14 months (*r*_24_ = 0.45, *p* = 0.029). In contrast, no significant correlations were observed between grasping slopes and subsequent language scores (*p*s > 0.281). Sitting and grasping slopes did not correlate with each other (*r*_24_ = -0.06, *p* = 0.761), but the current report will only focus on the relation between motor and language skills. Correlation results are summarized in **Table 2**.

**Table 2:** Correlations between motor and language skills.

Measure	1	2	3	4
(1) Grasping slope	-	-0.06	-0.15	-0.23
(2) Sitting slope		-	0.40^∗^	0.45^∗^
(3) CDI 10 months			-	0.59^∗∗^
(4) CDI 14 months				-


CDI, Communicative Development Inventories; Significant correlations are highlighted in bold. ^∗^*p* < 0.05, ^∗∗^*p* < 0.01.

### Regression Analyses

To complement our correlation findings, we examined whether the growth of sitting skills would predict subsequent language development above and beyond influences from concurrent motor skills. This question was addressed using two separate regression models with receptive language scores as outcome variables and sitting slope and concurrent motor skills (EMQ scores) as predictor variables. Due to the high variability in both CDI and sitting slope scores, robust regression was performed (Rousseeuw et al., 2015). At 10 months of age, sitting slope was a significant predictor above and beyond influences of 10 month EMQ scores, *t*(26) = 2.09, *p* = 0.046, *B* = 0.76, β = 0.39 [95% *CI* = (0.02, 0.75)]. Using a corresponding regression model at 14 months of age, sitting slope was again a significant predictor above and beyond influences of 14 month EMQ scores, *t*(21) = 3.21, *p* = 0.004, *B* = 0.54, β = 0.65 [95% *CI* = (0.04, 0.84)].

In addition to screening for outliers and using robust regression, analyses were also performed with three potentially influential observations removed from the data. This conservative approach confirms sitting slope as significant predictor of receptive vocabulary size at 10 months of age, *t*(23) = 2.21, *p* = 0.037, β = 0.43, [95% *CI* = (0.05, 0.78)]. However, the model fails to reach significance at 14 months of age (*p* = 0.40), potentially due to the overall smaller sample and lower statistical power at this age.

**FIGURE 2:**
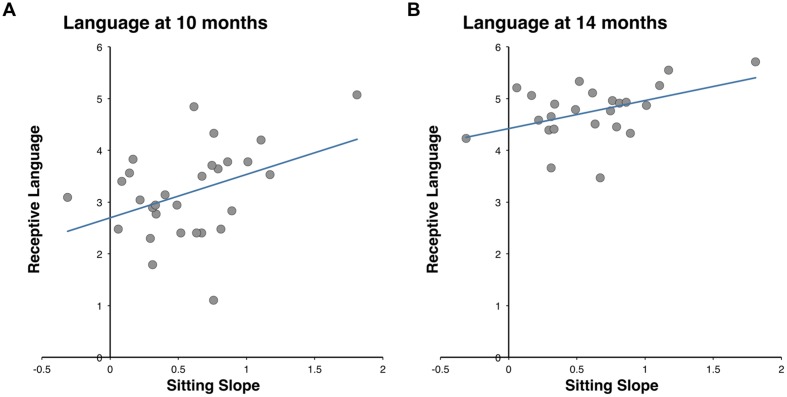
**Relation between sitting slopes (growth from 3–5 months) and receptive language vocabulary size (log transformed) at 10 months of age (A) and at 14 months of age (B)**.

Together, correlation and regression results suggest that the emergence of sitting skills between 3–5 months is related to receptive language development in the following months of life (**Figure 2**). The relation between sitting development and receptive language remains significant after controlling for concurrent motor skills assessed via parent report.

## Discussion

The goal of the current study was to longitudinally examine associations between early emerging motor skills and subsequent language development in typically developing infants. We hypothesized that the development of both reaching and sitting skills in early infancy would predict infants’ receptive vocabularies at 10 and 14 months of age. Our hypotheses were only partially supported. Results revealed a relation between language and sitting skills, but not between language and grasping skills. These findings have implications for our understanding of the interrelations among social, motor, and cognitive skills in infancy. In addition, our study demonstrates how direct observations of infants’ motor development can be collected remotely via videoconference.

### Relations between Motor and Language Development

The current findings confirm and expand prior results by longitudinally examining infant behavior before and during their acquisition of a new motor milestone. Previous studies have focused on infants’ mastery of a new motor skill and its relation to concurrent language development. For example, walking status around 10–14 months of age has been associated with larger vocabularies in both American and Chinese infants (Walle and Campos, 2014; He et al., 2015), and oral motor control around 21 months of age has been found to correlate positively with concurrent language skills (Alcock and Krawczyk, 2010). Others have reported predictive relations between earlier emerging motor skills and subsequent language development. For example, fine motor skills between 12 and 18 months of age have been found to predict expressive language at 36 months in infants at high familial risk for ASD (LeBarton and Iverson, 2013). The onset of independent sitting and walking have both been found to predict productive vocabulary sizes between 16 and 28 months (Oudgenoeg-Paz et al., 2012), and a large cohort study reported associations between motor skills at 18 months and *subsequent* language skills at 36 months of age (Wang et al., 2014). Our findings add to this growing evidence for motor-language associations by demonstrating a relation between the emergence of sitting skills around 3–5 months of age and subsequent language development at 10 and 14 months of age. However, the mechanism underlying this relation remains unknown.

### General Maturation vs. Developmental Cascades

At least two different theories predict associations between early motor skills and subsequent language development: the maturation hypothesis and the developmental cascades hypothesis. The maturation hypothesis suggests that motor advances are the result of general maturation processes that affect all domains of development equally (Gesell and Thompson, 1934). While this view does predict positive associations between motor skills and other domains of development, it also suggests that the relations between motor and language development should be bidirectional due to the underlying mechanism (maturation) being shared. However, previous findings did not observe a bidirectional relation between motor and language skills. For example, Wang et al. (2014) reported that motor skills at 18 months predict language at 36 months, but found no association between language skills at 18 months and motor skills at 36 months. Further, maturation would lead to general associations between motor and language development that are not limited to specific motor skills. In contrast, the current findings show a specific relation between sitting skills and language development, but no relation between grasping skills and language. These two observations suggest that general maturation alone may not be sufficient to explain the specific motor-language associations observed during development.

The developmental cascades hypothesis emphasizes the consequences following attainment of new motor skills as a driving force during development. Developmental cascades refer to the cumulative consequences of advances in one domain (e.g., motor skills) on later behaviors or abilities (Gottlieb, 1991; Fry and Hale, 1996; Masten and Cicchetti, 2010). Gaining a new skill leads to significant and long lasting changes in the child’s everyday experience by altering what kind of information is accessible and how others respond to the child. According to the developmental cascades theory, the onset of a new motor skill may provide infants with access to new learning opportunities associated with that motor skill. For example, being able to sit without support frees the hands for manual exploration of objects and enables learning about object features such as weight, texture, and function (Rochat and Goubet, 1995; Lederman and Klatzky, 2009). Sitting also frees the hands for the production of communicative gestures, which have been found to support language development (Iverson and Goldin-Meadow, 2005). Further, sitting changes the infants’ point-of-view, providing novel perceptual experiences and encouraging face-to-face exchanges with their caregivers. And finally, parents react to changes in infants’ abilities and adjust how they respond to the child (e.g., Karasik et al., 2014). In the context of the current study, the emergence of sitting skills at 3 months may initiate a developmental cascade by changing the child’s learning environment as described above: resulting in more opportunities for joint-attention, object-sharing, and object-labeling events that foster language development. Future research is needed to determine how exactly the emergence of independent sitting affects the child’s learning environment.

The developmental cascades theory would also predict that the onset of successful grasping has an impact on the child’s learning environment. However, the current study found no relation between the emergence of grasping and subsequent language skills. However, grasping might be indicative of language skills only following the onset of independent sitting. Put differently, sitting may act as a rate-limiting factor on the effects of grasping experiences. Indeed, studies with older children reported associations between fine motor sills and the subsequent cognitive abilities such as reading and math (e.g., Grissmer et al., 2010). Further, associations between motor and cognitive skills in early childhood seem to be mainly driven by fine manual control and visual perception skills (Davis et al., 2011). While fine motor skills are related to cognitive skills in mid-childhood, this relation may not be evident in early infancy as the hands are still needed to stabilize the body prior to the onset of independent sitting. This limits the experiences gained from independent grasping to structured exchanges where full trunk support is provided for the child. Following the onset of independent sitting, grasping and manual exploration skills may become more important predictors of language and cognitive skills.

### Remote Assessments of Early Motor Skills

The current study was the first to use of videoconferencing to collect behavioral data on infants’ sitting and grasping skills remotely. This approach allowed for higher-density data collection while reducing the overall burden on families in the study. Small sampling intervals are important to adequately capture the shape of developmental change over time (Adolph et al., 2008), and remote assessments may play an important role in future longitudinal studies in developmental psychology. However, there are some limitations of this method in general and of the current study in particular that need to be considered.

First, not all families have sufficiently fast access to the Internet in their own homes or do not own a camera-equipped computer, phone, or tablet to participate in the remote sessions. This may be one reason why the current study attracted mainly participants with relatively high levels of education. While the homogeneity of our sample does reduce the potential impacts of confounding factors (such as socioeconomic status), it greatly reduces the generalizability of our results. To encourage participation from a more economically diverse sample, we recommend that future studies should offer families incentives that compensate for the costs associated with participating in an online study (e.g., data plans, subscription fees).

Second, video chat only offers access to a small and static portion of the child’s home. The current study focused on pre-locomotor infants who could not yet crawl or walk. Consequently, infants remained in one position through the sitting and reaching assessments. With the onset of independent locomotion, infants may quickly move out of the view of the camera resulting in data loss. Consequently, remote assessments may not be feasible for all types of developmental research (e.g., to record unstructured parent-child interactions).

Finally, in our study, the parent acted as the experimenter to administer the grasping and sitting assessments. This resulted in increased assessment variability. To minimize variability in task administration, the current study used a realistic baby doll to demonstrate the procedure to all parents prior to their first session and an experimenter remained connected live with the parent during the study to comment and suggest changes as necessary.

## Conclusion

Motor skills play a critical role in early development and shape the child’s learning environment. The results reported here show associations between the emergence of sitting, but not grasping, skills around 3–5 months of age and subsequent receptive language at 10 and 14 months of age. These findings highlight the importance of early motor skills as an agent of change over time and suggest that the acquisition of a new motor skill may initiate developmental cascades that can influence subsequent language learning in typically developing infants. Further, the current study recorded infant behavior remotely via videoconference. Despite some limitations of this method, the minimal time burden on the part of the parent and the researcher as well as the minimal costs associated with videoconferencing demonstrate the value of this method for future research.

## Author Contributions

KL designed the study procedures and methods, collected the majority of the data, conducted all statistical analyses, interpreted the data, and wrote the first draft of the manuscript and edited subsequent versions. DV advised on study procedures, methods, and data interpretation, collected data, coded the majority of behavioral data, and edited the manuscript several times.

## Conflict of Interest Statement

The authors declare that the research was conducted in the absence of any commercial or financial relationships that could be construed as a potential conflict of interest.

## References

[B1] Adolph K. E. Young J. W. Robinson S. R. Gill-Alvarez F. (2008). What is the shape of developmental change? *Psychol. Rev.* 115 527–543. 10.1037/0033-295x.115.3.527 18729590 PMC2654330

[B2] Alcock K. J. Krawczyk K. (2010). Individual differences in language development: relationship with motor skill at 21 months. *Dev. Sci.* 13 677–691. 10.1111/j.1467-7687.2009.00924.x 20712734

[B3] Bhat A. N. Landa R. J. Galloway J. C. (2011). Current perspectives on motor functioning in infants, children, and adults with autism spectrum disorders. *Phys. Ther.* 91 1116–1129. 10.2522/ptj.20100294 21546566

[B4] Bornstein M. H. Hahn C. S. Suwalsky J. T. (2013). Physically developed and exploratory young infants contribute to their own long-term academic achievement. *Psychol. Sci.* 24 1906–1917. 10.1177/0956797613479974 23964000 PMC4151610

[B5] Bushnell E. W. Boudreau J. P. (1993). Motor development and the mind – the potential role of motor abilities as a determinant of aspects of perceptual development. *Child Dev.* 64 1005–1021. 10.1111/J.1467-8624.1993.Tb04184.X 8404253

[B6] Cashon C. H. Ha O. R. Allen C. L. Barna A. C. (2013). A U-shaped relation between sitting ability and upright face processing in infants. *Child Dev.* 84 802–809. 10.1111/cdev.12024 23199285 PMC3594454

[B7] Davis E. E. Pitchford N. J. Limback E. (2011). The interrelation between cognitive and motor development in typically developing children aged 4-11 years is underpinned by visual processing and fine manual control. *Br. J. Psychol.* 102 569–584. 10.1111/j.2044-8295.2011.02018.x 21752007

[B8] Fenson L. Marchman V. A. Thal D. J. Dale P. S. Reznick J. S. Bates E. (2006). *The MacArthur-Bates Communicative Development Inventories User’s Guide and Technical Manual*, 2nd Edn. Baltimore, MD: Brookes Publishing Company.

[B9] Fry A. F. Hale S. (1996). Processing speed, working memory, and fluid intelligence: Evidence for a developmental cascade. *Psychol. Sci.* 7 237–241. 10.1111/j.1467-9280.1996.tb00366.x

[B10] Gesell A. Thompson H. (1934). *Infant Behavior: Its Genesis and Growth*, 1st Edn. New York, PA: McGraw-Hill Book Company, Inc.

[B11] Gibson E. J. (1988). Exploratory behavior in the development of perceiving, acting and acquiring of knowledge. *Annu. Rev. Psychol.* 39 1–41. 10.1146/Annurev.Ps.39.020188.000245

[B12] Gottlieb G. (1991). Experiential canalization of behavioral development: theory. *Dev. Psychol.* 27 4–13. 10.1037/0012-1649.27.1.4

[B13] Grissmer D. Grimm K. J. Aiyer S. M. Murrah W. M. Steele J. S. (2010). Fine motor skills and early comprehension of the world: two new school readiness indicators. *Dev. Psychol.* 46 1008–1017. 10.1037/a0020104 20822219

[B14] Harbourne R. T. Lobo M. A. Karst G. M. Galloway J. C. (2013). Sit happens: Does sitting development perturb reaching development, or vice versa? *Infant Behav. Dev.* 36 438–450. 10.1016/j.infbeh.2013.03.011 23644424

[B15] He M. Walle E. A. Campos J. J. (2015). A cross-national investigation of the relationship between infant walking and language development. *Infancy* 20 283–305. 10.1111/infa.12071

[B16] Hill E. L. (2001). Non-specific nature of specific language impairment: a review of the literature with regard to concomitant motor impairments. *Int. J. Lang. Commun. Disord.* 36 149–171. 10.1080/13682820010019874 11344592

[B17] Iverson J. M. Goldin-Meadow S. (2005). Gesture paves the way for language development. *Psychol. Sci.* 16 367–371. 10.1111/j.0956-7976.2005.01542.x 15869695

[B18] Karasik L. B. Tamis-LeMonda C. S. Adolph K. E. (2014). Crawling and walking infants elicit different verbal responses from mothers. *Dev. Sci.* 17 388–395. 10.1111/desc.12129 24314018 PMC3997624

[B19] LeBarton E. S. Iverson J. M. (2013). Fine motor skill predicts expressive language in infant siblings of children with autism. *Dev. Sci.* 16 815–827. 10.1111/desc.12069 24118709 PMC3808875

[B20] Lederman S. J. Klatzky R. L. (2009). Haptic perception: a tutorial. *Attent. Percept. Psychophys.* 71 1439–1459. 10.3758/app.71.7.1439 19801605

[B21] Leonard H. C. Hill E. L. (2014). Review: the impact of motor development on typical and atypical social cognition and language: a systematic review. *Child Adolesc. Ment. Health* 19 163–170. 10.1111/camh.12055 32878369

[B22] Libertus K. (2010). *Object-Directed Action Experiences and their Effect on Cognitive and Social Development*, Doctor of Philosophy, Duke University, Durham, NC.

[B23] Libertus K. Joh A. S. Needham A. W. (2015). Motor training at 3 months affects object exploration 12 months later. *Dev. Sci.* 2015 1–9. 10.1111/desc.12370 PMC4916043 26689742

[B24] Libertus K. Landa R. J. (2013). The Early Motor Questionnaire (EMQ): a parental report measure of early motor development. *Infant Behav. Dev.* 36 833–842. 10.1016/j.infbeh.2013.09.007 24140841 PMC3858411

[B25] Libertus K. Landa R. J. (2014). Scaffolded reaching experiences encourage grasping activity in infants at high risk for Autism. *Front. Psychol.* 5:1071 10.3389/fpsyg.2014.01071 PMC4171992 25295021

[B26] Libertus K. Needham A. (2010). Teach to reach: the effects of active vs. passive reaching experiences on action and perception. *Vis. Res.* 50 2750–2757. 10.1016/j.visres.2010.09.001 20828580 PMC2991490

[B27] Libertus K. Needham A. (2011). Reaching experience increases face preference in 3-month-old infants. *Dev. Sci.* 14 1355–1364. 10.1111/j.1467-7687.2011.01084.x 22010895 PMC3888836

[B28] Libertus K. Needham A. (2014). Encouragement is nothing without control: factors influencing the development of reaching and face preference. *J. Motor Learn. Dev.* 2 16–27. 10.1123/jmld.2013-0019

[B29] Libertus K. Sheperd K. A. Ross S. W. Landa R. J. (2014). Limited fine motor and grasping skills in 6-month-old infants at high risk for Autism. *Child Dev.* 85 2218–2231. 10.1111/cdev.12262 24978128 PMC4236283

[B30] Lloyd M. MacDonald M. Lord C. (2013). Motor skills of toddlers with autism spectrum disorders. *Autism* 17 133–146. 10.1177/1362361311402230 21610184 PMC3188325

[B31] Masten A. S. Cicchetti D. (2010). Developmental cascades. *Dev. Psychopathol.* 22 491–495. 10.1017/S0954579410000222 20576173

[B32] Needham A. (2000). Improvements in object exploration skills may facilitate the development of object segregation in early Infancy. *J. Cogn. Dev.* 1 131–156. 10.1207/S15327647jcd010201

[B33] Needham A. Barrett T. Peterman K. (2002). A pick-me-up for infants’ exploratory skills: early simulated experiences reaching for objects using ’sticky mittens’ enhances young infants’ object exploration skills. *Infant Behav. Dev.* 25 279–295. 10.1016/S0163-6383(02)00097-8

[B34] Nickel L. R. Thatcher A. R. Keller F. Wozniak R. H. Iverson J. M. (2013). Posture development in infants at heightened versus low risk for autism spectrum disorders. *Infancy* 18 639–661. 10.1111/infa.12025 24027437 PMC3767458

[B35] Oudgenoeg-Paz O. Volman M. C. Leseman P. P. (2012). Attainment of sitting and walking predicts development of productive vocabulary between ages 16 and 28 months. *Infant Behav. Dev.* 35 733–736. 10.1016/j.infbeh.2012.07.010 22982273

[B36] Piaget J. (1952). *The Origins Of Intelligence in Children.* New York, NY: International Universities Press Inc.

[B37] Provost B. Lopez B. R. Heimerl S. (2007). A comparison of motor delays in young children: autism spectrum disorder, developmental delay, and developmental concerns. *J. Autism. Dev. Disord.* 37 321–328. 10.1007/s10803-006-0170-6 16868847

[B38] Rochat P. Goubet N. (1995). Development of sitting and reaching in 5-month-old to 6-month-old infants. *Infant Behav. Dev.* 18 53–68. 10.1016/0163-6383(95)90007-1

[B39] Rousseeuw P. Croux C. Todorov V. Ruckstuhl A. Salibian-Barrera M. Verbeke T. (2015). *Robustbase: Basic Robust Statistics. R Package Version 0.92-5*. Available at: http://CRAN.R-project.org/package = robustbase

[B40] Soska K. C. Adolph K. E. Johnson S. P. (2010). Systems in development: motor skill acquisition facilitates three-dimensional object completion. *Dev. Psychol.* 46 129–138. 10.1037/a0014618 20053012 PMC2805173

[B41] Teitelbaum P. Teitelbaum O. Nye J. Fryman J. Maurer R. G. (1998). Movement analysis in infancy may be useful for early diagnosis of autism. *Proc. Natl. Acad. Sci. U.S.A.* 95 13982–13987. 10.1073/pnas.95.23.13982 9811912 PMC25000

[B42] Walle E. A. Campos J. J. (2014). Infant language development is related to the acquisition of walking. *Dev. Psychol.* 50 336–348. 10.1037/a0033238 23750505

[B43] Wang M. V. Lekhal R. Aaro L. E. Schjolberg S. (2014). Co-occurring development of early childhood communication and motor skills: results from a population-based longitudinal study. *Child Care Health Dev.* 40 77–84. 10.1111/cch.12003 22970997

