# Supplementary material for: Sit to Talk: Relation between Motor Skills and Language Development in Infancy
Source: Front Psychol. 2016 Mar 31;7:475. doi: 10.3389/fpsyg.2016.00475 (PMC4815289; doi:10.3389/fpsyg.2016.00475)
Supplement: Supplementary file 1 [file Data_Sheet_1.PDF]

© Klaus Libertus Revision 1.6

Early Motor Questionnaire (EMQ)

Child's Lab ID: ____________________ Child's birthdate: Month:____ Day:____ Year:____

Chronological age: ______Months, ______Days Today's date: Month:____ Day:____ Year:____

Was your child born premature (<37 weeks gestation)? O Yes, at ______weeks of gestation O No, full term.

Relation to child: ____________________ Child's gender: O Male O Female

Do you have a child diagnosed with an Autism Spectrum Disorder? O Yes O No

Please read carefully before starting.

As you read each description of each behavior below, please indicate how certain you are whether your child already shows the described behavior or not. Please rate your certainty by circling one of the numbers in the right column. A 0 indicates that you are not certain whether your child shows this behavior or not. A -2 or 2 indicates that you are very certain and can recall a particular instance where your child showed this behavior.

**Sure that child does NOT show behavior** (e.g., you have seen your child fail when attempting this or a similar behavior)	**Child probably does NOT show behavior yet**	**Unsure whether child could do this or not** (please try to use this category seldom)	**Child probably shows this behavior**	**Sure that child shows this behavior and remember a particular instance**
-2	-1	0	1	2
You are sure that your child does not show this behavior yet.<br><br>*You can recall a particular instance where you child has failed this or a related behavior.*<br><br>*The behavior is not developmentally appropriate yet.*	*You cannot recall an instance where your child has attempted and failed this behavior, but your child does not show similar behaviors and you think he/she may not show this behavior yet.*	*You cannot recall an instance and you are not sure whether your child would show this behavior or not.*<br><br>*Please use this category sparingly.*	*You cannot recall a particular instance but your child shows a similar behavior.*<br><br>*Somebody (friend, nanny, daycare provider, other caretaker) has told you that your child shows this behavior*	*You have seen this behavior in your child.*<br><br>*You recall a particular instance when the behavior occurred.*<br><br>*Your child used to show this behavior but now shows more advanced behaviors (e.g., now walking instead of crawling)*

→ **When rating a behavior your child used to do but that is not developmentally appropriate anymore (e.g., crawling when the child is already walking alone) please rate this behavior as +2.**

This questionnaire covers ages 2-24 months, there are behaviors listed your child **may not yet show (circle -2)**, or used to show but **may not be evident any more (circle +2)**.

Sure that child does NOT show behavior<br>(e.g., you have seen your child fail when attempting this or a similar behavior)	Child probably does NOT show behavior yet	Unsure whether child could do this or not<br>(please try to use this category seldom)	Child probably shows this behavior	Sure that child shows this behavior and remember a particular instance
-2	-1	0	1	2

Section 1: Gross motor skills

(49 items)

In the following please think about your child's gross motor skills and motor control. These skills relate to how easily your child is able to control his or her own movements, orient, obtain toys, or move around the room.

→ Skills are organized by posture and increase in difficulty within each posture.

When held up against your shoulder, your child will					
01) snuggle in and rest at your body immediately?	-2	-1	0	1	2
02) hold head steady without support when looking around?	-2	-1	0	1	2
03) hold head steady while you bounce up and down	-2	-1	0	1	2
04) hold head steady while you walk or bend down	-2	-1	0	1	2
**When lying on his/her tummy, your child will**					
05) lift head slightly up from the ground and turn head to one side	-2	-1	0	1	2
06) lift head fully off the ground by pushing on his/her forearms	-2	-1	0	1	2
07) roll over to be on his/her back	-2	-1	0	1	2
**When lying on his/her back, your child will**					
08) move arms and legs vigorously (kicking and reaching movements)	-2	-1	0	1	2
09) hold on to your hands and pull herself/himself up to a sit without help	-2	-1	0	1	2
10) roll over to be on tummy	-2	-1	0	1	2
11) roll over to one side and push up into a crawling position	-2	-1	0	1	2
12) get up into a standing position by rolling to a side without help or aids	-2	-1	0	1	2
13) stand up without rolling to a side by sitting up and then moving forward	-2	-1	0	1	2
**When sitting on your lap with back support provided by you, your child will**					
14) hold his/her head up and steady when looking around the room	-2	-1	0	1	2
**When placed into a crawling position resting on hands and knees, your child will**					
15) shift weight to one arm and extend the other to reach, wave, or point	-2	-1	0	1	2
16) lift up bottom and remain in this position for a short time?	-2	-1	0	1	2
17) crawl forward for a few steps (3-5)?	-2	-1	0	1	2
**When placed into a sitting position on the floor, your child is able to**					
18) sit independently without support (hands lifted up)	-2	-1	0	1	2
19) use hands and legs to scoot forward on his/her bottom?	-2	-1	0	1	2
20) maintain a stable sitting position while turning head and torso to look around?	-2	-1	0	1	2
21) hold on to some furniture and pull into a standing position	-2	-1	0	1	2
22) shift into a crawling position and try to crawl forward	-2	-1	0	1	2

Sure that child does NOT show behavior (e.g., you have seen your child fail when attempting this or a similar behavior)	Child probably does NOT show behavior yet	Unsure whether child could do this or not (please try to use this category seldom)	Child probably shows this behavior	Sure that child shows this behavior and remember a particular instance
-2	-1	0	1	2

When placed into a standing position, your child will

23) bounce up and down slightly while holding on to you with both hands	-2	-1	0	1	2
24) take a few (wobbly) steps while holding on to you with one hand	-2	-1	0	1	2
25) stand alone for a few seconds without help	-2	-1	0	1	2
26) walk 4 or 5 steps independently with arms raised	-2	-1	0	1	2
27) is able to stand and toss a ball at the same time without loosing balance and falling over	-2	-1	0	1	2
28) squat down to pick up a toy from the ground	-2	-1	0	1	2

When placed in front of a flight of stairs, your child is able to

29) creep up the stairs independently?	-2	-1	0	1	2
30) walk up stairs (4-5 steps) with both hands held by a caregiver?	-2	-1	0	1	2
31) walk up stairs (4-5 steps) with one hand held by a caregiver?	-2	-1	0	1	2
32) walk up stairs alone while holding onto a wall or railing?	-2	-1	0	1	2
33) walk up stairs without aid? (4 or more steps)	-2	-1	0	1	2
34) creep down the stairs independently (feet first)?	-2	-1	0	1	2
35) walk down stairs without aid or help?	-2	-1	0	1	2

When moving around freely, your child will

36) run short distances around the room	-2	-1	0	1	2
37) run around the room making turns and stops without falling	-2	-1	0	1	2
38) kick a ball or small toy forward with one of his/her feet	-2	-1	0	1	2
39) stand on one foot when holding your hand (e.g., during dancing)	-2	-1	0	1	2
40) demonstrate walking on toes for a short time	-2	-1	0	1	2
41) jump in place with both feet up in the air (e.g., during dancing)	-2	-1	0	1	2
42) hop in place while balancing on one foot	-2	-1	0	1	2
43) jump down from boxes, small steps, or similar without falling	-2	-1	0	1	2

When walking down a hallway or small room, your child will

44) walk in a straight line for a few (4-5) steps with arms up	-2	-1	0	1	2
45) walk in a straight path without bumping into the walls using arms to balance	-2	-1	0	1	2
46) walk in a straight line with arms lowered and swinging freely	-2	-1	0	1	2

During free play or pretend play, you notice your child is able to

47) walk backwards for several (5 or more) steps?	-2	-1	0	1	2
48) jump forward over small obstacles such as a curb or box?	-2	-1	0	1	2
49) purposefully do a somersault?	-2	-1	0	1	2

Sure that child does NOT show behavior (e.g., you have seen your child fail when attempting this or a similar behavior)	Child probably does NOT show behavior yet	Unsure whether child could do this or not (please try to use this category seldom)	Child probably shows this behavior	Sure that child shows this behavior and remember a particular instance
-2	-1	0	1	2

Section 2: Fine motor skills

(48 items)

In the following please think about your child's fine motor skills such as reaching, grasping, orienting, and drawing. How easily can your child manipulate small objects and coordinate his/her hands?

→ Skills are organized by posture and increase in difficulty within each posture.

While observing your child lying on his/her back in a crib, baby gym, or on the floor, you notice your child

01) holding his/her hands close to the body in little fists occasionally?	-2	-1	0	1	2
02) tightly holding on to a toy placed into his/her hand?	-2	-1	0	1	2
03) bringing both hands together near the face, chest, or tummy?	-2	-1	0	1	2
04) opening up the fingers of each hand spontaneously?	-2	-1	0	1	2
05) spontaneously bringing one hand up to the mouth?	-2	-1	0	1	2
06) pulling on a string to obtain an object beyond reach?	-2	-1	0	1	2

When sitting on your lap or in a high chair while playing with toys, you notice your child is able to

07) successfully hold on to a small object such as a ring or stick?	-2	-1	0	1	2
08) reach for a toy with one hand by extending the arm and fingers?	-2	-1	0	1	2
09) successfully grasp a toy with one hand following a reach?	-2	-1	0	1	2
10) transfer toys from one hand to the other hand?	-2	-1	0	1	2
11) purposefully bang toys on the table or tray?	-2	-1	0	1	2
12) purposefully drop toys or throw them off the table?	-2	-1	0	1	2
13) manipulate one toy with both hands simultaneously?	-2	-1	0	1	2
14) place beads on a string without help?	-2	-1	0	1	2

When sitting without support on the floor or in a small chair, you notice your child

15) shaking small toys such as a rattle without loosing balance?	-2	-1	0	1	2
16) picking up small objects (e.g., cheerios) using index finger and thumb?	-2	-1	0	1	2
17) holding one toy in each hand and banging them together?	-2	-1	0	1	2
18) taking toys out of a box, bucket, or container?	-2	-1	0	1	2
19) putting toys into a box, bucket, or container?	-2	-1	0	1	2
20) stacking 3 or more blocks vertically?	-2	-1	0	1	2

When playing with a book or magazine, most of the time your child will

21) turn several pages at the same time?	-2	-1	0	1	2
22) turn one page at a time?	-2	-1	0	1	2

Sure that child does NOT show behavior (e.g., you have seen your child fail when attempting this or a similar behavior)	Child probably does NOT show behavior yet	Unsure whether child could do this or not (please try to use this category seldom)	Child probably shows this behavior	Sure that child shows this behavior and remember a particular instance
-2	-1	0	1	2

When drawing on a sheet of paper using a crayon or pen, your child will					
23) grip the crayon with a fist?	-2	-1	0	1	2
24) grip the crayon with thumb and index finger (right side down)?	-2	-1	0	1	2
25) make a mark on the paper in any direction?	-2	-1	0	1	2
26) mark vertical lines on the paper?	-2	-1	0	1	2
27) mark horizontal lines on the paper?	-2	-1	0	1	2
28) copy and reproduce simple drawings (e.g., circle) by a caregiver?	-2	-1	0	1	2
29) copy and reproduce multi-stroke drawings (e.g., square)?	-2	-1	0	1	2
30) copy and reproduce complex drawings (e.g., letters, triangle, cross)?	-2	-1	0	1	2
31) draw letters the right way round and proportional in size?	-2	-1	0	1	2
**When playing with a sheet of paper, your child can do the following with the paper**					
32) grasp the paper and pull or wrinkle it?	-2	-1	0	1	2
33) roll the paper in a tube shape?	-2	-1	0	1	2
34) fold the paper three times (any way)?	-2	-1	0	1	2
35) fold the paper in half two times to form a square?	-2	-1	0	1	2
**When playing with a shape sorter or piggy bank, you notice your child**					
36) is able to successfully place circular shapes into the sorter?	-2	-1	0	1	2
37) is able to place complex shapes (e.g., triangle) into the sorter?	-2	-1	0	1	2
38) rotates and successfully inserts small objects (e.g., coins)?	-2	-1	0	1	2
**When playing with building blocks, you notice your child will**					
39) stack block towers of 6 blocks or more?	-2	-1	0	1	2
40) add a block to a tall tower without knocking it over?	-2	-1	0	1	2
41) copy you or other children by building a similar tower?	-2	-1	0	1	2
**When playing with activity books or color books, your child occasionally will**					
42) fill in color areas while staying inside the object's boundaries?	-2	-1	0	1	2
43) connect lines in a maze or draw by numbers game?	-2	-1	0	1	2
**When getting dressed or undressed, your child sometimes is able to**					
44) open shoelaces by pulling?	-2	-1	0	1	2
45) open Velcro latches by pulling?	-2	-1	0	1	2
46) open zippers by pulling?	-2	-1	0	1	2
47) open buttons?	-2	-1	0	1	2
48) close a zipper or buttons by himself/herself?	-2	-1	0	1	2

Sure that child does NOT show behavior (e.g., you have seen your child fail when attempting this or a similar behavior)	Child probably does NOT show behavior yet	Unsure whether child could do this or not (please try to use this category seldom)	Child probably shows this behavior	Sure that child shows this behavior and remember a particular instance
-2	-1	0	1	2

Section 3: Perception Action

(31 items)

In the following please think about your child's visual and receptive skills. Can your child make out small details on pictures? Does your child orient to sounds and respond to stimulation easily?

→ Skills are organized by posture and increase in difficulty within each posture.

While lying on his/her back in a crib, baby gym, or on the floor, your child sometimes will					
01) fixate on objects that are moved close to your child's eyes?	-2	-1	0	1	2
02) turn the head all the way to one side (90°) to follow your face?	-2	-1	0	1	2
03) notice his/her own hands and look at them for some time?	-2	-1	0	1	2
04) swat at toys hanging from a baby gym or car seat?	-2	-1	0	1	2

While sitting on your lap or fully supported in a high chair or car seat, you have noticed your child					
05) follow a person or object by turning his/her head slightly?	-2	-1	0	1	2
06) turn the head from side to side (180°) to follow something interesting?	-2	-1	0	1	2
07) shift eye gaze back and forth between your face and an object?	-2	-1	0	1	2
08) focus on a far away object (e.g., toy across the room)?	-2	-1	0	1	2
09) orient to noises and visually search for the cause of the noise?	-2	-1	0	1	2
10) extend his/her arms towards an object that is close by?	-2	-1	0	1	2
11) pull on a string or cloth to obtain a connected object?	-2	-1	0	1	2

When your child is sitting on the floor on his/her own without support, your child will					
12) pull an object to reveal another object that was hidden underneath?	-2	-1	0	1	2
13) find a hidden object when given multiple choices to search?	-2	-1	0	1	2
14) turn cups right side up during play?	-2	-1	0	1	2
15) sometimes use objects functionally and appropriately on him/herself (e.g., comb own hair with comb, eat with spoon)?	-2	-1	0	1	2
16) sometimes use objects functionally and appropriately on others (e.g., comb your hair, feed puppet)?	-2	-1	0	1	2

When playing with your child sitting at a table or in a high chair with tray attached, your child will					
17) open and close a book using two hands?	-2	-1	0	1	2
18) touch pictures in a book and vocalize about them?	-2	-1	0	1	2
19) turn cups right side up during play?	-2	-1	0	1	2
20) nest 2 or 3 nesting cups or containers by putting one inside another?	-2	-1	0	1	2
21) nest 4 or more nesting cups or toys correctly?	-2	-1	0	1	2

When playing with a wooden puzzle or form board, you child is able to					
22) insert simple, rounded shapes correctly into the puzzle?	-2	-1	0	1	2
23) insert shapes with edges (e.g., triangle) correctly into the puzzle?	-2	-1	0	1	2

Sure that child does NOT show behavior (e.g., you have seen your child fail when attempting this or a similar behavior)	Child probably does NOT show behavior yet	Unsure whether child could do this or not (please try to use this category seldom)	Child probably shows this behavior	Sure that child shows this behavior and remember a particular instance
-2	-1	0	1	2

When being asked to find or show an item, your child is able to

24) find items of pairs (e.g., where is the other shoe, sock, glove)?	-2	-1	0	1	2
25) find identical items (e.g., this is your spoon, where is my spoon)?	-2	-1	0	1	2
26) match pictures in a picture book (e.g., where is the other dog)?	-2	-1	0	1	2
27) match letters (e.g., pointing out same letters)?	-2	-1	0	1	2

When cleaning up after play or sorting during play, your child can when asked to

28) sort toys by category (e.g., blocks in one box, puppets in another)?	-2	-1	0	1	2
29) sort toys by shape, size, or color?	-2	-1	0	1	2

When reading a book or looking at a picture in a newspaper or photo book, your child will

30) point to the same item or person across multiple pictures?	-2	-1	0	1	2
31) look to where you point?	-2	-1	0	1	2

Comments and concerns:

Reference:

Libertus, K., & Landa, R. J. (2013). The Early Motor Questionnaire (EMQ): A parental report measure of early motor development. *Infant Behavior and Development, 36*(4), 833-842.

http://www.ncbi.nlm.nih.gov/pubmed/24140841
https://www.sciencedirect.com/science/article/pii/S0163638313000969

Klaus.Libertus@gmail.com
